# The how’s and what’s of vaccine reactogenicity

**DOI:** 10.1038/s41541-019-0132-6

**Published:** 2019-09-24

**Authors:** Caroline Hervé, Béatrice Laupèze, Giuseppe Del Giudice, Arnaud M. Didierlaurent, Fernanda Tavares Da Silva

**Affiliations:** 1 GSK, Wavre, Belgium; 2 GSK, Siena, Italy

**Keywords:** Signs and symptoms, Vaccines

## Abstract

Reactogenicity represents the physical manifestation of the inflammatory response to vaccination, and can include injection-site pain, redness, swelling or induration at the injection site, as well as systemic symptoms, such as fever, myalgia, or headache. The experience of symptoms following vaccination can lead to needle fear, long-term negative attitudes and non-compliant behaviours, which undermine the public health impact of vaccination. This review presents current knowledge on the potential causes of reactogenicity, and how host characteristics, vaccine administration and composition factors can influence the development and perception of reactogenicity. The intent is to provide an overview of reactogenicity after vaccination to help the vaccine community, including healthcare professionals, in maintaining confidence in vaccines by promoting vaccination, setting expectations for vaccinees about what might occur after vaccination and reducing anxiety by managing the vaccination setting.

## Reactogenicity after vaccination

Reactogenicity refers to a subset of reactions that occur soon after vaccination, and are a physical manifestation of the inflammatory response to vaccination. In clinical trials, information on expected signs and symptoms after vaccination is actively sought (or ‘solicited’). These symptoms may include pain, redness, swelling or induration for injected vaccines, and systemic symptoms, such as fever, myalgia, headache, or rash. The broader term ‘safety’ profile refers to all adverse events (AEs) that could potentially be caused/triggered or worsened at any time after vaccination, and includes AEs, such as anaphylactic reactions, diseases diagnosed after vaccination and autoimmune events.

Thousands of vaccines are administered every day and the vast majority induce few symptoms in the recipient. Those symptoms that do occur early after vaccination are usually mild and self-limiting and rarely have serious medical consequences. Nevertheless, understanding the range of symptoms that vaccination might cause is important for the person receiving the vaccine, for caregivers/decision-makers deciding to accept vaccination for others, and for the healthcare professionals who recommends and administers vaccines. Reactogenicity can contribute to a person’s willingness to be vaccinated. If a vaccine is perceived as too reactogenic, the subject may refuse further doses, or the healthcare professional may elect not to recommend it, leading to incomplete protection of the individual and low vaccine coverage in the population. Maintaining high vaccine coverage is critical to the success of vaccination programs.

The majority of vaccines available today are administered parenterally via injection. Knowledge about the symptoms that can occur after vaccination can be used by healthcare professionals to prepare patients on what to expect after vaccination, potentially leading to improved compliance and higher acceptance of subsequent doses. This review presents current knowledge on what potentially causes reactogenicity, and how host characteristics, vaccine composition and vaccine administration techniques influence the development and perception of reactogenicity. We summarise current recommendations for pain mitigation at the time of vaccination, including the benefits and risks of using antipyretics to prevent symptoms.

## Evaluation of vaccine reactogenicity and safety

The current attitude regarding the benefits versus the risks of vaccination puts a large emphasis on safety, because vaccines are usually given to healthy populations who may receive no immediate health benefit, particularly when the incidence of the target infectious disease is low. Vaccine reactogenicity and safety is assessed at all points of the vaccine development process, from preclinical toxicology studies using cell cultures and animal models, to rigorous assessment in clinical studies and post-licensure pharmacovigilance.^1^ Following licensure, safety remains of prime concern; ongoing surveillance evaluates vaccine safety in large populations under real-world settings. Fundamental to this process is the contribution of the Brighton Collaboration, a network whose aim is to establish clear case definitions for individual events following vaccination, and to thereby ensure high-quality data collection and reporting consistency.^2,3^

Pre-clinical evaluation investigating injection-site reactions and the toxicological profile of vaccines is a prerequisite to the initiation of clinical trials in which safety and efficacy are established. However, while preclinical studies are critical to identify signals that would prevent their use in humans, evaluation in the early phase of clinical development is needed to confirm the safety profile of the vaccine in humans. Systematic collection of safety data in clinical trials follows guidelines set out by competent regulatory agencies, such as the European Medicines Agency, the US Food and Drug Administration and World Health Organization (WHO). Authorities also advise on the size of the safety database needed to provide reasonable assurance that the reactogenicity and safety profile of the vaccine is acceptable.

In clinical trials, reactogenicity is measured by collecting sets of injection site and systemic signs and symptoms that are solicited from study participants over a specified time after vaccination. The types of symptoms collected and the period for which they are collected depend on the vaccine being investigated and the objectives of individual studies. Reactions that are solicited (in which participants are actively questioned about the occurrence of each symptom) are usually reported at higher frequencies than the same symptoms when participants are asked to report them spontaneously (unsolicited symptoms).

While some signs can be objectively measured (body temperature, redness, swelling, heart rate), other symptoms are non-specific and subjective, and are perceived differently depending on a range of factors occurring at the time. These factors can include mood, presence of other medical conditions or symptoms, climate, individual perceptions of pain, etc. Effects of symptoms on an individual’s physical functioning and quality of life, reduced work efficiency or use of healthcare resources or medications are also difficult to assess quantitatively. Furthermore, many illnesses and general conditions cause the same signs and symptoms (for example, fever, headache, fatigue), making it challenging to determine what is, and what is not caused by vaccination. A double-blind study conducted in 581 twin pairs in Finland illustrated this problem.^4^ One twin received measles-mumps-rubella vaccine (MMR) followed by placebo injection 3 weeks later, while the second twin received the injections in the reverse order. The study showed that during the time of peak incidence of fever, 88% of low-grade fever, 24% of moderate fever and 7% of high fever episodes were not caused by the vaccine. These observations highlight the importance of an appropriate control group (such as placebo) in clinical trials in order to be able to untangle which proportion of signs or symptoms might be caused by vaccination.

After authorization, vaccine safety is monitored through pharmacovigilance activities conducted by the manufacturer, regulatory authorities and independent researchers, such as review of spontaneous reports of AEs received from healthcare professionals, lay persons and regulatory agencies world-wide. Investigation of specific AEs of interest may continue in targeted safety studies conducted post-licensure. When needed, the prescribing information is updated to reflect the latest safety data.

## What causes reactogenicity?

**Fig. 1:**
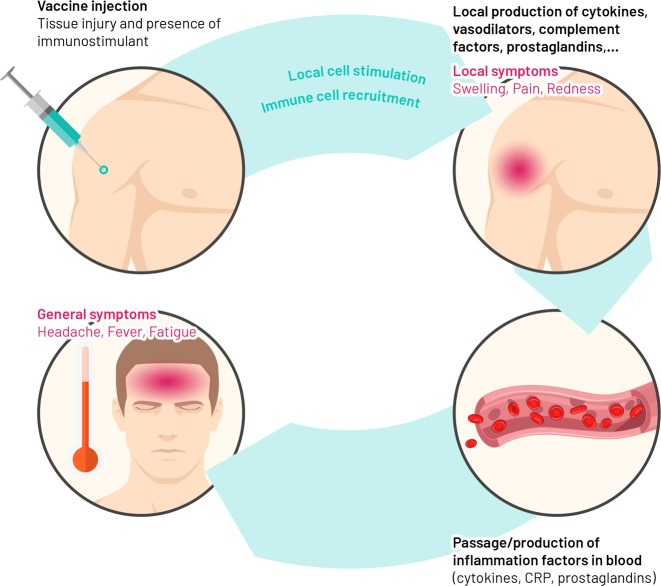
Summary of expected biological mechanisms underlying the development of reactogenicity symptoms. Vaccine antigens and immune enhancers (as adjuvants) injected into the muscle are recognised by the body as potential pathogens and/or danger signals. This recognition leads to the stimulation of local cells, followed by the recruitment of blood immune cells to the local site and the production of different soluble factors including vasodilators and cytokines, which may trigger the development of signs and symptoms of local inflammation (pain, redness and swelling). The passage of some of those factors in the bloodstream, as well as the production of other systemic factors by immune blood cells or distant organs (e.g. liver), may contribute to the development of general symptoms (fever, myalgia, headache etc) in the vaccinee. *CRP* C-reactive protein

Vaccines contain antigens that induce an immune response capable of providing specific protection from disease. Individual vaccine antigens induce innate immune responses that may differ qualitatively or quantitatively according to the vaccine composition, but that induce a good adaptive immune response. After entering the body, vaccine antigens are recognised as potential pathogens by conserved pathogen-associated molecular patterns (PAMPs) or damage-associated molecular patterns (DAMPs), pattern-recognition receptors (PRRs) including Toll-like receptors (TLR)^5^ that are found on local or peripheral circulating immune cells (e.g. monocytes and macrophages) and on resident stromal cells.^5,6^ The transcription of many target genes is induced in these cells, resulting in the synthesis and release of pyrogenic cytokines (i.e. interleukin [IL]-1, IL-6, tumour necrosis factor-alpha [TNF-α], and prostaglandin-E2) in the bloodstream, that mimics the response to natural infection. Once stimulated, the immune system sets off a complex series of innate immune events that can include phagocytosis, release of inflammatory mediators including chemokines and cytokines, activation of complement, and cellular recruitment. These phenomena are crucial for triggering strong antigen-specific acquired immune responses necessary for protection against disease. These same inflammatory events may also lead to the development of signs and symptoms of injection-site inflammation (pain, redness and swelling) in the vaccinated individual (Fig. 1). Mediators and products of inflammation in the circulation can affect other body systems to cause systemic side-effects (such as fever, fatigue, and headache). Balancing the beneficial versus the detrimental effects of these inflammatory events is necessary to keep reactogenicity at clinically acceptable levels.

The following section provides a review on the current understanding of the mechanisms related to reactogenicity with a focus on vaccines given via the intramuscular route. Effects of the route of immunization on reactogenicity are discussed later in this review.

### What causes injection-site symptoms?

#### General concept

All vaccines share the capacity to activate PRRs that will lead to the production of different mediators. PRRs are expressed by immune cells, including monocytes, macrophages, mast cells and dendritic cells, and resident stromal cells, such as keratinocytes and skeletal muscle cells. Resident cells, in particular macrophages and mast cells, are key target cells that initiate the response within minutes of vaccination, releasing pro-inflammatory cytokines, chemokines, effectors of the complement cascade (C3a and C5a) and vasodilators, including vasoactive amines and bradykinin.^7^

**Fig. 2:**
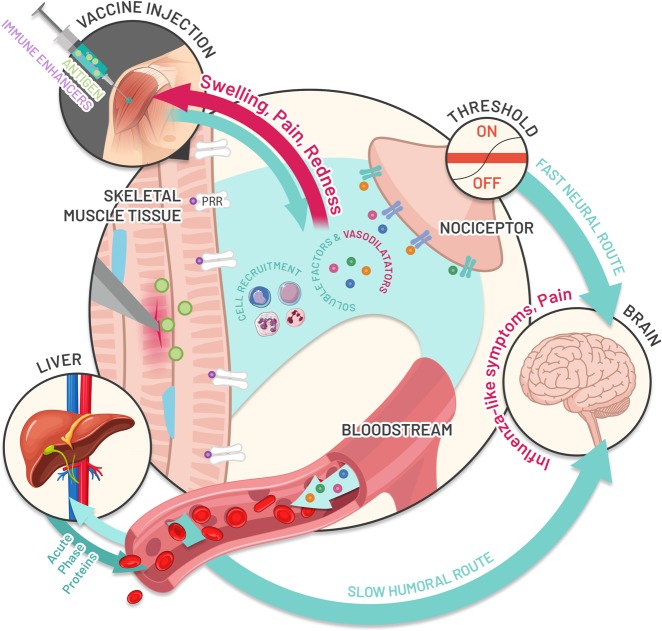
Hypothesised link between the innate immune response induced by vaccination and reactogenicity. Upon vaccination, inflammation is triggered by innate immune activation of pattern-recognition receptors (PRR) including Toll-like receptors (TLRs) that recognize and bind antigens (green circle in skeletal muscle) and potential immune enhancers (purple circle in skeletal muscle) present in the vaccine formulation. Resident immune cells, mast cells, monocytes and macrophages are activated within minutes of injection and release soluble factors (proinflammatory cytokines, chemokines, effectors of the complement cascade) and vasodilators, that allow cell recruitment from blood but also lead to the development of redness and swelling symptoms. These newly recruited immune cells, mainly composed of blood-born neutrophils, monocytes and T lymphocytes, also contribute to pain sensation by releasing soluble factors, such as cytokines, prostaglandins or ATP, that can directly interact with local sensory receptors called nociceptors and cause pain through the fast neural route if the threshold is reached. Once produced, cytokines act both locally in autocrine and paracrine manners, and may act systemically at distant organs, leading to the production of C-reactive protein and other acute phase proteins by the liver. Several immune-to-brain signaling pathways may propagate an inflammatory response to the central nervous system after peripheral activation of the innate immune system (slow humoral route), leading to the development of fever and sickness behaviours

Vasodilators and the chemokine gradient promote cell recruitment from blood, but also lead to the development of redness and swelling (Fig. 1). Blood-borne neutrophils, monocytes and lymphocytes adhere to the vessel walls and accumulate at the site of injury via extravasation. These immune cells may contribute to peripheral nociceptive sensitisation by releasing soluble factors, such as cytokines, prostaglandins or ATP, and interacting directly with nociceptors (sensory neurons that respond to potentially damaging stimuli) to cause pain if the pain threshold is reached. Pain sensation is transmitted through fast-conducting myelinated neurons (the fast neural pathway) (Fig. 2).^8^

#### Vaccine-specific immune profile at the injection site

Adjuvants are immunostimulants that enhance the immune response to the antigen, and usually increase reactogenicity compared to inactivated vaccines or purified antigens without adjuvant.^9,10^ Aluminium salts were the first adjuvant ever licensed for use in human vaccines. Examples of newer adjuvants in use today include virosomes, oil-in-water emulsions (such as MF59, which contains squalene and is used in seasonal and pandemic influenza vaccines), immunostimulants, such as MPL (3-*O*-desacyl-4 monophosphoryl lipid A), CpG 1018 (a CpG-containing oligonucleotide sequence used in a hepatitis B vaccine) and proprietary combinations of immunostimulants referred to as Adjuvant Systems. The composition and actions of new adjuvants has been recently reviewed.^11–13^

Not all vaccines require the addition of adjuvants (self-adjuvanted). Most of the studies describing local innate immune responses in pre-clinical models involved adjuvanted vaccines, but it is assumed that all types of vaccines trigger a similar response, with kinetics and amplitude influenced by the nature of vaccine components (live/non-live/type of adjuvant) and the site of administration.

After injection of vaccines containing an Adjuvant System, chemokines and cytokines are readily detected in mouse muscle as early as 3 h post-injection.^14–16^ Most cytokines and chemokines in muscle injected with adjuvanted vaccine decrease rapidly within 24 h after delivery, and reach baseline after 72 h. The expression of pro-inflammatory molecules is not only restricted to muscle. Lymph nodes that drain adjuvanted vaccine-injected muscle show higher cytokine and chemokine expression compared with lymph nodes that drain muscle that received phosphate-buffered saline or aluminium salt,^17^ or protein antigen alone. For AS03 (an oil-in-water emulsion with squalene and alpha-tocopherol [Vitamin E] mixed in the oil phase), the cytokine response in draining lymph nodes is detected a few hours after injection, similarly to injected muscle, suggesting that AS03 may drain into the node and act directly, along with antigen-loaded cells that arrive from injected muscle.^17^

In terms of innate cells, neutrophils and monocytes are the first cells to infiltrate antigen-exposed tissues. In mice, they are recruited to muscle as early as 3–6 h after injection of either MF59^16^ or AS01 (QS-21, *Quillaja saponaria* Molina: fraction 21. QS-21 is licensed by GSK from Antigenics LLC, a wholly owned subsidiary of Agenus Inc., a Delaware, USA corporation, and MPL in a liposome-based formulation),^17^ while other immune cells, including dendritic cells, eosinophils, natural killer cells and lymphocytes are recruited later,^15–17^ which likely leads to cross-talk between cells to orchestrate innate immune responses. Neutrophils and monocytes return to steady-state levels in adjuvant-injected muscle after 5–7 days.^15,17^ Collectively, these data show that local inflammation in the muscle and draining lymph node after immunisation is a transient physiological event coinciding with the duration of solicited injection-site symptoms described in clinical trials after vaccination.

### What causes systemic symptoms after intramuscular or subcutaneous vaccination?

#### General concept

The mediators and products of inflammation at a localised site in the body may spill into the circulation and can affect other body systems causing systemic side-effects. These systemic pyrogenic factors, along with PAMPs, DAMPs and adhering monocytes, trigger cross-talk between the immune response and the central nervous system via receptors on the vagus nerve, at the blood-brain barrier and perhaps within circumventricular organs.^18,19^ Within the brain, the coupled induction of the inducible enzymes cyclooxygenase-2 and microsomal prostaglandin E synthase-1 by these signal molecules results in elevated intracerebral levels of prostaglandin E2, the critical terminal mediator of raised body temperature and other systemic symptoms, such as headache, myalgia and chills (or ‘sickness syndrome’).^20–22^ Intracerebral prostaglandin E2 finally activates neuronal circuits which adjust autonomic and behavioural responses, such as peripheral vasoconstriction, metabolic heat production, shivering (‘chills’) or warmth-seeking behaviour, thereby causing a rise in body temperature.

#### Vaccine systemic immune profile and link to reactogenicity

The main step in the development of systemic symptoms after vaccination is thought to be the presence of inflammatory markers in the bloodstream, which signal at the blood-brain barrier level and induce influenza-like symptoms.

Several publications have reported the nature and kinetics of inflammatory mediators induced by vaccines and their potential association with reactogenicity symptoms. In healthy adults that received the hepatitis B virus surface Antigen (HBsAg) combined with different Adjuvant Systems (AS01, AS03, AS04 [MPL adsorbed onto aluminium hydroxide or aluminium phosphate] or aluminium salts), all Adjuvant Systems induced transient systemic innate responses, including IL-6 and C-reactive protein (CRP), mostly peaking at 24 h post administration and subsiding to baseline within 1 to 3 days.^23^ A follow-up study showed that some of these inflammatory markers were more associated with systemic than injection-site reactogenicity (www.clinicaltrials.gov NCT01777295).^24^ The study could not identify specific markers that could be directly correlated with, or predict symptoms.

Similar increases in circulating cytokines within hours and days after vaccination were reported for the adjuvanted human papillomavirus and influenza vaccines.^25–27^ In one study looking at the AS03-adjuvanted H1N1 vaccine (H1N1/AS03), a higher number of a specific B cell subset at baseline prior to vaccination was proposed as a biomarker correlating with specific AEs, although a causal relationship with these cells and specific symptoms and/or inflammatory markers induced by the vaccine remains to be demonstrated.^26^

Adjuvanted vaccine formulations are not the only formulations to induce transient systemic inflammation. Several publications describe the early systemic immune response induced in humans after the administration of non-adjuvanted vaccines (reviewed in Lim et al.,^28^). All of the clinical studies consistently described a slight and short-lived increase in inflammatory mediators in blood following vaccination, in particular, an increase in CRP and IL-6. The level and kinetics of the inflammatory markers depended on the type of vaccine used and the population studied.^28^

A mild and transient increase in circulating IL-6 and TNF-α, with peak responses 1 day post vaccination was shown in pregnant women vaccinated with non-adjuvanted trivalent inactivated seasonal influenza vaccine.^29^ An increase in a specific set of immune genes was also observed 1 day post vaccination in subjects that received a live-attenuated yellow fever vaccine.^30^ At this time point, the genes belonging to TLR and interferon (IFN)-signalling pathways were differentially expressed in subjects with systemic AEs as compared to subjects without AEs, although the differences in gene expression were not statistically different. An association was also found between specific systemic symptoms (myalgia, headache) and stem cell factors and CD100 (cluster of differentiation 100) pathways known to play a regulatory role in central nervous system functions as well as immune responses, thus reinforcing the probable involvement of immune-to-brain signalling^30^ in the development of systemic reactogenicity. Finally, an association between several systemic cytokines and chemokines, and fever, myalgia, chills and headache was reported 1 day after injection in subjects who had received a recombinant vesicular stomatitis virus-vectored Zaire Ebola vaccine.^31^

### Summary

It is a common belief that an injection-site reaction to a vaccine is a predictive sign of a desirable vaccine response (‘no pain, no gain’ concept). However limited data either support or disprove this concept.^32^ In the hepatitis B study comparing different adjuvants,^23^ the magnitude of inflammatory responses paralleled the magnitude of adaptive immune responses and the overall incidence of reactogenicity symptoms. The more potent adjuvants (AS01 and AS03) were capable of activating an early IFN-signalling pathway, which was confirmed in the blood of subjects the day after receiving H1N1/AS03.^26^ However, despite parallel associations of reactogenicity and adaptive responses with early innate responses, no predictive association was demonstrated between reactogenicity and the adaptive response, which suggests that the ‘no pain, no gain’ concept may not be valid, at least at the individual level.^32^

Vaccines, irrespective of their composition, induce some level of inflammation at the injection site within the first hours after their administration which is likely to contribute to causing pain, redness and swelling symptoms. Release of pyrogenic factors into the systemic circulation is thought to stimulate a cascade of immune and nervous system cross-talk that can lead to systemic ‘influenza-like’ symptoms including raised body temperature. There is growing evidence of general associations between systemic inflammatory mediators and systemic symptoms after vaccination. However, no single biomarker of systemic reactogenicity has been identified, but rather a composite of biomarkers; in particular, IL-6, CRP, and for highly immunogenic products, the IFN-signalling pathway, appear to be linked to systemic reactogenicity. To date, it is unknown whether the specific molecular pathways that cause symptoms are independent from pathways involved in the generation of antigen-specific response. This knowledge is required for the design of less reactogenic vaccines, or targeted strategies aimed at reducing the severity of symptoms. The discovery of potential biomarkers of reactogenicity is being actively pursued through collaborations within academy and industry such as the BIOVACSAFE consortium.^33^

## Factors that can influence reactogenicity

**Fig. 3:**
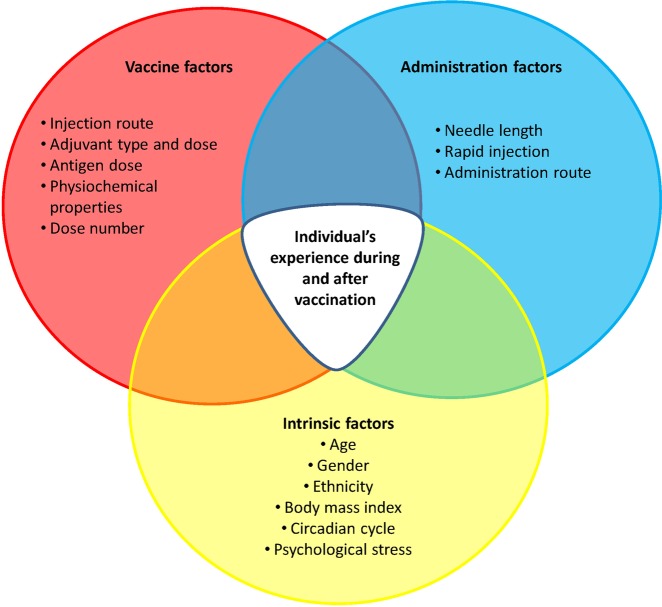
Summary of vaccination and host factors that can influence reactogenicity. As a general concept, all the conditions that can influence the immune, the endocrine or the hormonal systems of the host (intrinsic factors), all the conditions that can increase tissue stress (administration factors) and the components that activate innate immunity contained in the vaccines (vaccine factors) could theoretically impact the incidence and severity of local and general symptoms induced after vaccination

Extrinsic and intrinsic factors can impact the reactogenicity profile, tolerability and immunogenicity of vaccines in a given individual (Fig. 3). They include host characteristics, such as age, gender, race/ethnicity, body mass, general health and pre-existing immunity, and vaccine administration and composition factors, such as route and site of administration, injection technique, type of antigen, vaccine formulation, and type of adjuvant. The sections below provide some specific examples of the concepts being discussed.

### Host characteristics that can influence reactogenicity

#### Age

Physiological functions of the immune and nervous systems evolve throughout life. These changes have implications for defence against infectious diseases at different ages, and can also influence susceptibility to adverse reactions to vaccination. While infants and toddlers tend to exhibit fewer injection-site reactions after vaccination than adults, they are more prone to experience fever episodes due to vaccination or other co-incidental infections.^4^

Reporting rates of AEs following immunisation increase during childhood and adolescence as the immune system matures. Reporting rates of AEs reduce during adult life, possibly due to higher tolerance to pain and illness symptoms gained with life experience and/or the waning of innate immune defence mechanisms. The latter is supported by the observation that older people display lower systemic levels of IL-6, IL-10 and CRP after vaccination,^34^ which could contribute to their tendency to report fewer systemic AEs, in particular fever.

#### Gender

Compared to men, women tend to experience higher incidences of injection site, but not systemic symptoms after vaccination,^35–38^ and may experience higher rates of immediate hypersensitivity reactions.^39^ Possible explanations could be related to genetic or hormonal differences.^36^ For example, anatomical differences in skin thickness, blood flow and nervous system structure between men and women may favour the development of injection-site inflammation in women.^40^ Further, sex hormones have been shown to influence immune responses and cytokine levels, with androgens and high doses of oestrogens being immunosuppressive.^41,42^

#### Ethnicity

Little is known about whether ethnicity influences vaccine reactogenicity because comparisons are confounded by the effect of cultural influences on the interpretation of symptoms and the propensity to report them. Differences in circulating inflammatory cytokine levels and genetic polymorphisms between ethnic groups have been observed.^43–45^ However, the interpretation and comparison of such studies are complicated by the multitude of circumstances, life-events, and socio-cultural aspects that influence how symptoms are perceived and reported.^46^

#### Psychological/physical stressors and circadian cycles

Stress in various forms and circadian cycles are known to influence the immune system^47^ and in particular the inflammatory response. Subjects experiencing acute or chronic stressors are characterised by high levels of circulating pro-inflammatory cytokines.^48^ During the circadian cycle, pro-inflammatory hormones and cytokines are synchronised to facilitate the initiation of adaptive immune responses in lymph nodes during nocturnal sleep, while during daytime activity, anti-inflammatory signals, hormones and cytokines appear to support immediate effector functions.^49^ Consequently, it has been suggested that the timing of vaccine or drug delivery and the level of stress could influence immunogenicity and tolerability of the intervention.^49–51^ However, to date, no studies have clearly assessed the impact of those factors on the development of vaccine-solicited AEs.

#### Overweight/obesity

Obesity has been demonstrated to be associated with low-level chronic inflammation.^52^ However, studies suggest that increases in reactogenicity in the overweight population are most likely due to vaccine administration technique and not to body mass itself.^53–55^ In a study of overweight/obese participants and in normal weight controls, the frequency of injection-site and systemic reactions after trivalent influenza vaccine was statistically similar between groups.^53^ Other studies have suggested that infants and young children with higher body mass are more likely to have injection-site reactions from acellular pertussis vaccines, possibly due to inadvertent subcutaneous administration.^54^ A correlation was identified between body mass index and reactogenicity using the New Zealand meningococcal serogroup B vaccine.^55^ However, this association was no longer present when the results were controlled for vaccinator, suggesting individual injection techniques were responsible for this observation.

#### Pre-existing immunity (pre-existing before vaccination and vaccine-induced)

With few exceptions,^56^ natural immunity has not been found to affect the overall safety profile of vaccines targeting the said pathogen. Efforts to correlate pre-vaccination antibody titres with the incidence of injection-site or systemic reactions following vaccination have not identified consistent associations.^57–59^

Some vaccines cause increased injection-site and general reactions after repeated doses. For example, injection-site and systemic symptoms increase with successive doses of whole-cell and acellular pertussis-containing vaccines.^60^ Varicella-containing vaccines (monovalent and combined measles-mumps-rubella and varicella, MMRV) cause increased rates of injection-site reactions in those children receiving the varicella antigen for the second time.^61^ Five–six-year-old children receiving MMRV were more likely to have injection-site reactions including pain, redness, and swelling if they had previously received a dose of MMRV as opposed to MMR.^56^ The underlying mechanisms are not fully understood and may be related to pre-existing immunity induced by priming to antigen or another vaccine component, mediated by T cells which can be quickly recruited to the site of inflammation, or to local innate immune memory responses, or to the age of the vaccinee at booster dose administration. For some vaccines, such as hepatitis A vaccine, adverse reactions become less frequent after additional doses.^62,63^

Periodic booster doses with tetanus, diphtheria and pertussis vaccines are recommended in adults, as well as annual influenza vaccination and 5-yearly pneumococcal polysaccharide vaccination. In adults vaccinated with polysaccharide pneumococcal vaccines, increased symptoms after re-vaccination has not been consistently observed, although increased reactogenicity compared to the first dose seemed to occur if the interval between doses was below 5 years.^59^ Reactogenicity can increase with the number of tetanus vaccines administered (either alone or combined with diphtheria toxoid and acellular pertussis components), which can be reduced by decreasing the dose of toxoid administered to adults.^64^ Hypersensitivity reactions have been reported after repeated immunization with tetanus toxoid. These are thought to be caused by antigen-antibody complex formations as a result of vaccination.^65^ Influenza vaccines are administered annually to various populations but the impact of repeated annual vaccination on reactogenicity is poorly defined. Three studies showed no differences in injection-site or systemic symptoms in adults and children who had received up to five previous influenza vaccinations,^66–68^ whereas one study showed an increased rate in local reactions after repeated seasonal influenza vaccination in children.^69^

Few studies have estimated the risk of recurrence of specific AEs after re-vaccination (reviewed by Zafack et al.^70^). In children who experienced extensive limb swelling after vaccination with acellular pertussis vaccines, up to 78% experienced extensive limb swelling at the next dose.^70^ In preterm infants who experienced apnoea after the first vaccine dose, only 18% had a recurrence rated as severe as the initial episode. Overall, the risk of a recurrence of AEs at the next dose in people who have had AEs after vaccination appears to be low.^70^

### Vaccine characteristics that can influence reactogenicity

#### Route, site and method of vaccine administration

Administering vaccine into a different route to that recommended can adversely affect reactogenicity. For injected vaccines, the depth of the injection has a strong influence on reactogenicity, with deep injections (intramuscular) usually associated with fewer injection-site symptoms than subcutaneous or intradermal injections,^71–73^ probably because skeletal muscle has fewer pain fibres compared with skin and subcutaneous tissue. A clinical trial of MMR and varicella vaccines showed substantially higher rates of solicited injection-site reactions in the groups that received subcutaneous injection.^74^ Despite good evidence of the better tolerability of intramuscular injection, sub-cutaneous rather than the intra-muscular injection is preferred in some countries.^71^ Skin inflammatory reactions following intradermal vaccination against influenza are likely to be immunologically mediated since they have been less frequently observed in immunocompromised subjects receiving the intradermal vaccine.^75^ Orally administered vaccines are usually very well tolerated, but are occasionally followed by gastrointestinal symptoms, such as vomiting or diarrhoea.

The intranasal route is attractive for prevention of mucosal infections,^76^ but currently, only one vaccine (live-attenuated influenza vaccine) is available for use by the intranasal route. Potential side-effects of intra-nasal influenza vaccine include nasal congestion, wheezing, headache, vomiting, muscle aches, fever, sore throat and cough.^77^

Other vaccine administration routes under investigation include non-invasive approaches, such as plant-based oral vaccines,^78^ and transdermal methods, such as microneedle patches.^79^ AEs specific to these administration routes, such as skin irritation in the case of microneedle devices or AEs following plant-based oral vaccines, are yet to be quantified.

The reactogenicity profile of a vaccine can vary significantly between individual vaccine administrators.^54,55^ Injection site symptoms are minimised when the needle is inserted at 90° to the skin using a needle size of appropriate length to ensure administration into the correct tissue. Additionally, acute injection-site pain is decreased when a rapid injection is performed,^80^ because slow injection causes prolonged tissue distension and damage from lateral movement of the needle, further exacerbated by muscle contraction and movement in response to the administration.^81^

Sterile abscesses (caused by irritation and not infection) can rarely occur after vaccination, and are possibly a result of a hypersensitivity reaction to a vaccine component.^82^

#### Vaccine composition

Vaccines are composed of whole pathogens or antigen(s) variously combined with adjuvants, preservatives, stabilisers, other excipients and remnants of the manufacturing process. All of these ingredients, as well as the volume of liquid to be injected, can potentially cause symptoms on injection. Injection of volumes larger than 0.8 ml is reported to cause more pain than when the volume injected is smaller.^83^

Live-attenuated vaccines contain whole pathogens that are designed to replicate within the body inducing mild infection, potentially inducing mild disease symptoms.^84^ Fever and rash may occur after measles vaccine, peaking 5–12 days after vaccination which coincides with the peak in viral replication. Live-attenuated vaccines contain an array of antigens and innate immune triggers which usually makes them immunogenic, but potentially more reactogenic than combinations of purified antigens.

Killed vaccines also contain whole organisms that have been killed through chemical or physical means, and are usually also highly immunogenic and potentially reactogenic.^84^ Subunit vaccines containing one or several purified components are usually less reactogenic than whole-cell vaccines (killed or live-attenuated) but may also be less immunogenic, thus requiring the addition of an adjuvant to restore the immune triggers. Adjuvanted vaccines have been linked to higher rates of injection-site pain and systemic symptoms, such as myalgia or headache than unadjuvanted controls, but unsolicited AEs, febrile seizures, potential immune-mediated diseases and new onset of chronic diseases occur at similar rates.^9^

Physical and chemical properties of the vaccine formulation, such as pH, viscosity and charge of the vaccine substance can impact reactogenicity.^85^ For example, in some studies but not all, pain on injection of *MMR-II* (Merck & Co.), and pain in the days following *MMR-II* injection, is significantly higher than for *Priorix* (GSK) even though they contain the same antigens.^80,86–88^ Both vaccines were well tolerated and if a difference exists, it could be due to differences in pH between the two formulations.^80^ Osmolarity does not appear to greatly influence reactogenicity.^89^

#### Antigen and adjuvant dose and type

Increasing the antigen dose may induce stronger immune responses but may also cause higher levels of reactogenicity.^90^ Because of the immune-enhancing effects of adjuvants, their addition to a vaccine can lower the dose of antigen needed to induce similar or enhanced immune responses compared to higher amounts of antigen administered alone. While the amount of antigen may decrease, the addition of adjuvant in these vaccines generally increases reactogenicity as compared to unadjuvanted vaccine containing the full antigen dose.^91^ As observed for non-adjuvanted vaccines, the majority of reactions after vaccination with adjuvanted vaccines are of short duration, and consistent with kinetics of the inflammatory response described in animal studies.^9,15,92,93^

The different adjuvants that are included in vaccines differ in their mode of action and ability to stimulate the immune system, and consequently, their contribution to reactogenicity symptoms may vary. There are only few studies comparing different adjuvants in humans. A recent study comparing the response to HBsAg formulated in different Adjuvant Systems showed that the level of reactogenicity was associated with the ability of Adjuvant Systems to stimulate an innate response and to promote higher HBs-specific responses.^94^ In this study, the highest local reactogenicity occurred in the group that received AS01.^94^ AS01 is included in the recently licensed recombinant herpes zoster vaccine (*RZV*, GSK). While highly efficacious in preventing herpes zoster in older adults,^95^ pain at the injection site was commonly reported in up to 88.4% of vaccinees, depending on age, versus up to 14.4% of placebo recipients.^95^ The reactogenicity profile was characterised by mild to moderate reactions generally of short duration (median duration of pain was 2.0 days in adults^96^), and was consistent with the profile of other AS01-adjuvanted vaccines and the kinetics of the inflammatory response induced by RZV in animal studies.^15^

#### Combinations and co-administration

Combining different antigens in the same vaccine or co-administration of vaccines provides significant advantages in ensuring optimal vaccine coverage and compliance. Co-administration of vaccines can result in higher reactogenicity overall, although this is not a general rule. For example, post-marketing studies showed that the risk of fever and convulsions was higher when MMRV was used for the first dose, compared with MMR + varicella co-administered separately.^97,98^

## Symptom management

**Fig. 4:**
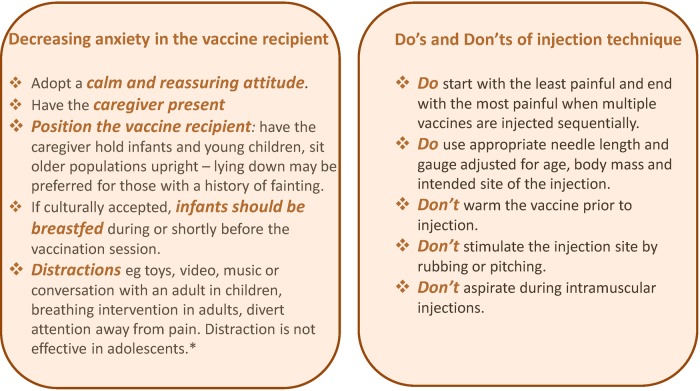
Practical recommendations to decrease side-effects that may occur at the time of injection: adapted from the World Health Organization recommendations.^99^ *There are no additional evidence-based, age-specific recommendations available for adolescents beyond the general measures recommended for all age groups

Pain and distress at the time of vaccination are important clinical issues for individuals of all ages undergoing injection. Not addressing pain at the time of vaccination can engender vaccine hesitancy and may impact on future health-seeking behaviour and healthcare decisions. There are numerous interventions that can help reduce both the immediate and delayed side-effects that may result from vaccination, should they occur. In 2015, WHO published recommendations for pain mitigation at the time of vaccination, aiming at decreasing anxiety in the vaccine recipient and reminding healthcare professionals about good injection practices including the use of appropriate needle length and selection of appropriate anatomical location for injection (Fig. 4).^99^

Topical anaesthetics used in children before injection can also reduce immediate pain and needle distress. WHO does not recommend the routine use of topical anaesthetics because of their high cost, lack of availability in many settings and the prolonged onset of action. Routine use of topical anaesthetics is recommended for 0- to 12-year-old children in Canada in view of evidence of reduced distress in young children when they are used, and to reduce long-term development of needle fears and future non-compliant behaviours.^100^ Novel active distraction techniques that use virtual reality can be used effectively to reduce pain and anxiety in paediatric patients undergoing vaccination.^101^

Redness and mild swelling are not usually associated with significant discomfort and usually require no treatment or can be managed with local application of ice. Pain in the days following injection, and systemic symptoms, such as headache, fever, and myalgia can cause substantial discomfort that may be mitigated by the administration of paracetamol (acetaminophen), aspirin or anti-inflammatories such as ibuprofen.

### Can prophylactic medications prevent symptoms?

A meta-analysis of 13 randomised controlled trials concluded that while prophylactic antipyretics significantly reduced injection-site and systemic symptoms after vaccination, their use was associated with reduced antibody responses to most vaccine antigens.^102^ A reduction in the response to pneumococcal conjugate vaccines was the most consistently observed impact, although children who had received prophylactic antipyretics still achieved seroprotective levels of antibodies to all of the administered antigens.^102^ Another recent review concluded that the timing of antipyretic administration was key, because no effect on the antibody response was seen when antipyretics were given as a treatment for symptoms (rather than for prevention of symptoms) after vaccination (Table 1).^103^ Thus, the use of ibuprofen and paracetamol in children to treat symptoms arising after vaccination appears to be effective, without impacting the immune response to vaccination.

**Table 1:** Impact of paracetamol versus ibuprofen on reactogenicity and immunogenicity

	Impact on reactogenicity	Impact on antibody response	Reference
*Oral paracetamol* ^a^			
Administered prophylactically at the time of vaccination and within the following 24 h	↓ Tenderness	↓ 5/13 pneumococcal serotypes, hepatitis, diphtheria, tetanus. Lower impact after dose 2.	^105, 106, 112, 113^
	↓ Fever		
	↓ Swelling		
	↓ Pain		
Administered therapeutically	↓ Fever	No impact.	
	↓ Pain		
*Oral ibuprofen* ^b^			
Administered prophylactically at the time of vaccination and within the following 24 h	Limited impact	↓ pertussis toxin, tetanus. No impact after the second dose.	^103, 112^
Administered therapeutically	↓ Fever	No impact.	
	↓ Pain		

^a^One publication in older adults (mean ages 73–75 years). Results showed no impact on antibody levels and limited impact on symptoms

^b^No data in older adults

Of few studies conducted in adults, neither low-dose aspirin, nor prophylactic paracetamol impacted the response to influenza vaccines.^104,105^ However, one study showed that prophylactic (but not therapeutic) paracetamol significantly reduced the adult immune response to a hepatitis B vaccine.^106^ Nevertheless, antibody levels in each group in this study were high, and all subjects had seroprotective antibody levels after the second dose.

Because of the need to balance reactogenicity with the potential effect of prophylactic paracetamol on immunogenicity, there are only few vaccines for which prophylactic administration of paracetamol to prevent symptoms is currently actively recommended, usually in situations when different pyrogenic vaccines are co-administered, which results in an additive effect on the incidence of fever. Currently this includes co-administration of *Infanrix hexa* (GSK) and pneumococcal conjugate vaccines, *Infanrix hexa* with MMRV vaccines, and of the 4-component meningococcal serogroup B vaccine with routine vaccines.^107,108^ These recommendations were made based on clinical trial data showing that co-administration increased the risk of fever in infants by a factor considered to be clinically significant, which could be reduced by administering paracetamol at, or soon after vaccination.

## The role of the healthcare professional

Healthcare professionals are at the frontline of vaccine provision, often responsible for recommending and administering vaccines, and for managing those seeking medical advice for symptoms after vaccination. Healthcare professionals involved in vaccine delivery are in an ideal position to promote the benefits of vaccination, particularly in settings where the fear of side-effects plays a role in influencing decisions to vaccinate,^109,110^ setting expectations for vaccinees about what might occur post vaccination, and reducing anxiety by managing the vaccination setting.^111^ Correct immunisation procedures including appropriate needle length and site of administration can impact reactogenicity, potentially reducing pain on injection and the occurrence of other delayed symptoms. Reporting AEs, should they occur, even if they are already in the prescribing information, is an important mechanism by which healthcare professionals contribute to the continuous monitoring of vaccine safety.

## Conclusion—maintaining confidence in vaccines

Reactogenicity symptoms are an outworking of the expected immune response that occurs in response to vaccination. However, the experience of symptoms is influenced by a multitude of factors, many of which can be alleviated by educating vaccine recipients, providing an appropriate environment for vaccination, and using good injection methods. Healthcare professionals play an integral role in these endeavours.

While we know that a certain level of inflammation is needed to trigger an effective adaptive immune response, we do not yet know how to quantify that level, or predict how this translates into reactogenicity. New technologies including systems biology are providing new insights into the early immune response to vaccination.^33^ This, and potential identification of biomarkers linked to reactogenicity, will allow further understanding of the links between immunogenicity and reactogenicity. In the future this may allow development of less reactogenic vaccines, or even identification of which individuals are likely to experience more severe symptoms after vaccination.

## Data sharing

Data sharing is not applicable to this article as this is a review paper and no new datasets were generated or analysed in the publication.
